# Predictors of return to work among women with long-term neck/shoulder and/or back pain: A 1-year prospective study

**DOI:** 10.1371/journal.pone.0260490

**Published:** 2021-11-23

**Authors:** Mamunur Rashid, Marja-Leena Kristofferzon, Annika Nilsson

**Affiliations:** 1 Department of Public Health and Sports Sciences, Faculty of Health and Occupational Studies, University of Gävle, Gävle, Sweden; 2 Department of Caring Sciences, Faculty of Health and Occupational Studies, University of Gävle, Gävle, Sweden; University of Minho, PORTUGAL

## Abstract

**Background:**

Sick leave due to musculoskeletal pain, particularly in the neck/shoulders and back, is one of the major public health problems in Western countries such as Sweden. The aim of this study was to identify predictors of return to work (RTW) among women on sick leave due to long-term neck/shoulder and/or back pain.

**Methods:**

This was a prospective cohort study with a 1-year follow-up. The study participants were recruited from a local Swedish Social Insurance Agency register and had all been on sick leave for ≥ 1 month due to long-term (≥ 3 months) neck/shoulder and/or back pain. Data on predictors and outcome were collected using a self-administered questionnaire. A total of 208 women aged 23–64 years were included at baseline, and 141 responded at the 1-year follow-up. Cluster analyses were performed to identify one predictor from each cluster for use in the regression model.

**Results:**

At the 1-year follow-up, 94 of the 141 women had RTW and 47 had not. Women who engaged in more coping through increasing behavioral activities (OR: 1.14, 95% CI: 1.03–1.25) and those who more strongly believed they would return to the same work within 6 months (OR: 1.22, 95% CI: 1.10–1.37) had an increased probability of RTW. Receiving more social support outside work (OR: 0.50, 95% CI: 0.28–0.92) decreased the odds of RTW at the 1-year follow-up.

**Conclusions:**

Behavioral activities, beliefs about returning to the same work, and social support outside work were predictors of RTW at the 1-year follow-up. Healthcare professionals should consider these predictors in their efforts to prevent prolonged sick leave and to promote RTW in this population.

## Introduction

Work is an important part of life and has a fundamental role in physical health and psychosocial well-being [1]. Working life may be interrupted by musculoskeletal pain (MSP) that can lead workers to take sick leave [2]. MSP occurs predominantly in the neck/shoulders and back and more frequently in women than in men, and has been considered the second most frequent reason for sick leave in Western countries [2–6]. According to a report from the Swedish Social Insurance Agency, 61% of the Swedish population on sick leave for MSP are women [7]. Individuals on sick leave for MSP may experience positive or negative consequences of sick leave [8], depending on how they relate to their sick role during sick leave periods [8, 9]. The experiences of women on long-term sick leave may differ; some experience hopelessness and lack of motivation, whereas others may make plans for future work and seek support [10]. Being on long-term sick leave per se has been considered a predictor of future poor physical health, low psychosocial well-being, and reduced work ability [11]. Further, sick leave results in medical expenses, workers’ compensation, and productivity loss in Western countries, including Sweden [12, 13]. Considering the enormous economic cost, there is a need to examine factors that may obstruct or promote return to work (RTW) among women on sick leave for long-term MSP.

Ways of assessing RTW include work status, time until the suspension of time-loss benefits, and time until claim closure [14, 15]. The measures used vary with the population studied as well as across societies and countries, which have different healthcare systems and workers’ compensation regulations [14–16]. In the present study, RTW was assessed in terms of work status; that is, whether individuals were working or not, and the extent of the work. RTW following MSP is a multifaceted process that is not merely dependent on physical health and/or ability; rather, the process of RTW in this regard involves an individual’s resources, such as recovery beliefs and coping strategies. These resources are related to gradually increasing one’s work ability in order to be able to cope with work demands and life events such as stress and pain [17].

Several potential factors related to RTW among individuals with MSP have been studied previously. For example, psychological factors such as recovery beliefs, anxiety, depression, locus of control, and health-related quality of life have been shown to be associated with RTW among people with MSP [18–21]. Studies have also shown that RTW may be affected by pain-related behaviors such as pain intensity and fear-avoidance beliefs, and work-related factors such as job stress and job satisfaction [21, 22]. One study found that age, gender, motivation, coping, and general health status were important for RTW on the individual level [23]. Earlier research has also shown that individuals’ belief in their ability to work in the future is a predictor of RTW [24, 25], and that increased work ability per se is an important predictor for RTW among women with pain in the neck/shoulder and/or back [26]. Furthermore, social support from work and outside work influences recovery from long-term MSP [27], which may have a significant role in RTW [28]. Several studies have reported that social support outside work, such as close relationships and supportive social environment, is associated with reduced MSP. This may increase work ability, which could, in turn, contribute to RTW [26, 29]. Social support either from work or outside work was also found to be a positive indicator in the process of RTW [30, 31]. Finally, one study found that social support from a partner relationship might not be related to RTW among female workers on sick leave [32]. It seems that existing previous studies regarding social support and RTW have given mixed results, depending on the study population and measurement of social support.

Most of the previous studies on predictors of RTW among individuals with MSP have been conducted on workers in general, or only on male workers, and have focused on acute and sub-acute pain or non-specific low back pain [19–24, 28]. A previous systematic review [33] aimed at summarizing prognostic factors related to RTW among people with long-term neck/shoulder or back pain suggested that perceived health, recovery beliefs and work ability may be predictive of RTW. However, the authors concluded that their findings were based on only a few studies, and none of the studies in the review focused solely on women [33]. Factors associated with RTW may vary between men and women even if they are in the same line of work [34]. Moreover, women still have the highest proportion of sick leave for long-term MSP [7]. Because being on sick leave for long-term MSP is a problem from an individual, social and financial perspective, it is important to identify predictors of RTW that could be considered in rehabilitation for this population [35]. Thus, the aim of the present study was to identify predictors of RTW among women on sick leave for long-term neck/shoulder and/or back pain.

## Methods

### Study design and settings

This was a prospective cohort study with a 1-year follow-up. The study participants were sampled from a local Swedish Social Insurance Agency register which covered all people receiving sick leave benefits in Central and Northern Sweden. The periods of data collection were the spring of 2016 (baseline) and the spring of 2017 (follow-up).

### Study sample

The participants were selected by the Swedish Social Insurance Agency based on their medical certificate, which had been issued by their primary healthcare or hospital physician. Before the selection procedure, two of the authors (MLK and AN) instructed personnel at the Swedish Social Insurance Agency on how to select participants. To qualify for inclusion in the study, a participant had to be a woman aged 18–65 years, on ≥ 50% sick leave from her usual employment (i.e., she could be working part-time), and on sick leave for ≥ 1 month due to long-term neck/shoulder and/or back pain that had lasted for ≥ 3 months. Neck/shoulder and/or back pain were classified to the following diagnostic codes from version 10 of the International Classification of Diseases: M53.1 (cervicobrachial syndrome), M54.2 (cervicalgia), M54.4 (lumbago with sciatica), M54.5 (low back pain), M54.9 (dorsalgia unspecified), M75.8 (other shoulder lesions), M75.9 (shoulder lesion, unspecified), and M79.1 (myalgia). The code for myalgia (M79.1) was included because myalgia pain can spread to neck, shoulders, and back; for example, trapezius myalgia is characterized by acute or persistent neck/shoulder pain. Because the specific cause of MSP is often uncertain, many diagnostic codes are used for this population. The diagnostic codes were selected based on a previous study [36] and discussions with the Swedish Social Insurance Agency. Understanding the Swedish language was also required for the participants to complete the questionnaire. Women were excluded from the study if they had been diagnosed with rheumatoid arthritis, multiple sclerosis, stroke, cancer, Parkinson’s disease, bipolar disease, or schizophrenia, or were pregnant. These diseases, disorders, and conditions were chosen as exclusion criteria because individuals affected by them require different types of interventions and hence may have a different RTW process [37]. In addition, women who had early retirement (i.e., before 65 years of age) were not included in the study. The project was approved by the Regional Ethical Review Board in Uppsala, Sweden (Reg. no. 2.3.2-2015/548).

### Data collection

An initial invitation letter and a self-administered questionnaire including eight instruments were sent to the participants by the Swedish Social Insurance Agency. Several demographic variables such as age, cohabitation, number of children, education, years in the workforce, type of work, stress in the last 6 months, life-long pain duration, and physical activity were included in the questionnaire. In addition, a pain figure was included to collect information on the location of pain on the body [38]. Participants who agreed to take part in the study returned the questionnaire along with a signed informed consent form. To increase the response rate, two reminders were sent about two weeks apart. At the 1-year follow-up, the same questionnaire was sent to the baseline participants, this time including two additional background questions meant to detect RTW status.

### Candidate predictors

Based on a systematic review [33] and previous empirical research [19, 20, 39] on factors associated with RTW among people with MSP, the candidate predictors of RTW presented below were considered in this study.

The Coping Strategies Questionnaire [40] consists of eight subscales. In the present study, two subscales, “increase behavioral activity” and “ignore sensations”, were used to assess: (i) coping through increasing behavioral activities such as leisure activities, reading, and socialization; and (ii) coping by ignoring sensations, for example by relaxing, thinking pleasant thoughts, and praying [40]. These two subscales were chosen because they are commonly used in MSP patients [41, 42] and represent both cognitive and behavioral coping processes. The sub-scale “pain catastrophizing” was not selected for the present study because we did not measure RTW in terms of any direct pain-related outcome such as reduction in pain and disability [43]. Each scale was measured using six items rated on a 7-point Likert scale (0 = never; 6 = always). An index of each scale was obtained by calculating the sum of all scores of the six items, such that higher values represented more frequent use of the coping strategies. Cronbach’s α was 0.86 for each subscale [44].

Self-efficacy was measured using the General Self-Efficacy scale [45], which consists of 10 items rated on a 4-point Likert scale (1 = not true; 4 = completely true). Total scores on the GSE range from 10 to 40 points, with higher values representing greater self-efficacy. Cronbach’s α for this scale was 0.92 [44].

Sense of coherence was measured using a short version of the Sense of Coherence scale [46], which consists of 13 items rated on a 7-point scale (1 = never; 7 = very often). The total scores range from 13 to 91 points, with higher scores indicating greater sense of coherence. Cronbach’s α for this scale was 0.84 [44].

Physical activity was assessed using a single question: “How often do you exercise regularly for at least 30 minutes, e.g., walking, jogging, swimming, cycling, or walking in the garden?” Respondents answered the question by selecting one of four alternatives: 0 days/week, 1–3 days/week, 4–5 days/week or 6–7 days/week.

Beliefs about returning to the same work within 6 months were also assessed using a single question: “Do you believe you will return to the same work within 6 months?” Respondents answered on a 10-point Likert scale (1 = highly unlikely to return to the same work; 10 = highly likely to return to the same work).

Pain intensity was measured using three items from the Multidimensional Pain Inventory [47]: (i) How much pain are you experiencing right now? (ii) How much pain have you experienced on average during the past week? (iii) How much do you suffer from your pain? The participants rated each item on a 7-point Likert scale (0 = no pain; 6 = extreme pain). An index was created by calculating an average value of the items, with higher values indicating higher pain intensity. Cronbach’s α for this scale was 0.76 [44].

Social support outside work was measured using the social support subscale from the Multidimensional Pain Inventory, which consists of three items [47]: (i) When you are experiencing pain, how much support or help do you get from the people closest to you (family or friends)?, (ii) How concerned are your friends and family about the pain you experience?, (iii) How much consideration do your friends and family members show for your pain? Respondents rated the items on a 7-point scale (0 = not at all; 6 = very much). An index was created by calculating an average value of the items, with higher values indicating higher social support. Cronbach’s α for this scale was 0.60.

Depression was assessed using the Hospital Anxiety and Depression Scale [48], which consists of 14 items rated on a 4-point Likert scale; all of the even-numbered items form a depression subscale. Total scores on this subscale range from 0 to 21 points, with higher values indicating greater depression. Although the scale has two subscales (anxiety and depression), depression was chosen for this study because depression is more common in women than in men [49]. In addition, depression was found to be negatively correlated with well-being in the same population in a previous study, but this was not the case for anxiety [44]. Cronbach’s α for depression subscale was 0.91 [44].

The Demand Control Support Questionnaire [50] was used to measure job strain. This consists of four subscales: psychosocial demands (5 items), skills discretion (2 items), decision authority (4 items), and social support (6 items). For each item, responses were made on a 4-point Likert scale ranging from 1 (strongly agree) to 4 (strongly disagree). To capture job strain, an index was constructed for each of the psychological demands, skills discretion, and decision authority subscale. Skills discretion and decision authority were then merged into one scale called decision latitude. Afterwards, a job strain score was created by calculating the ratio between psychological demands and decision latitude, with higher values representing higher job strain [51]. Cronbach’s α for this was 0.57 [44].

Life-long pain duration was assessed using the single question: “How long have you been experiencing pain?” The response was reported in months.

### Outcome measure

Information on RTW was gathered at the 1-year follow-up using two questions: “Are you working right now?” and “To what extent are you working?”. RTW status was considered a dichotomous variable. If participants worked > 50% of their extent of employment at baseline, they were categorized as RTW; otherwise, they were categorized as not RTW (NRTW).

### Statistical analysis

Descriptive statistics for the demographic variables are presented as proportions, means, and standard deviations. Difference in means of RTW and NRTW by baseline characteristics and study variables were evaluated with a chi-squared test for binary variables, Fisher’s exact test for nominal and ordinal variables, and an independent t-test for numerical variables. Scatterplots showed that all variables were approximately normally distributed, and there were no outliers in the data. An attrition analysis was performed between participants and dropouts at the 1-year follow-up considering age, life-long pain duration, pain intensity, work ability, and well-being.

Because the number of participants limited the number of predictors that could be included in the prediction model [52], we had to select a subset of predictors among the candidates. To reduce the risk of modeling spurious relationships, the selection of predictors was made without empirically verifying their relationship with the outcome RTW [53, 54].

To reduce the number of predictors, the following steps were carried out. First, a hierarchical cluster analysis was performed on all candidate predictors using squared Euclidean distance and average linkage between clusters [53–55]. Second, one predictor from each cluster was chosen on the basis of (i) a formal statistical test which showed the relative dispersion of its values in the within-cluster sample, and (ii) the importance of previous findings in relation to RTW in this regard. Finally, multiple logistic regression was performed to estimate the association between the selected predictors and RTW. Age was controlled for in the adjusted analysis. Nagelkerke’s *R*^2^ was used to measure the overall predictive ability of the selected predictors of RTW in the logistic regression. The level of significance was set at *p* < 0.05. All data analyses were performed using version 24 of the IBM SPSS statistical software package.

## Results

**Fig 1:**
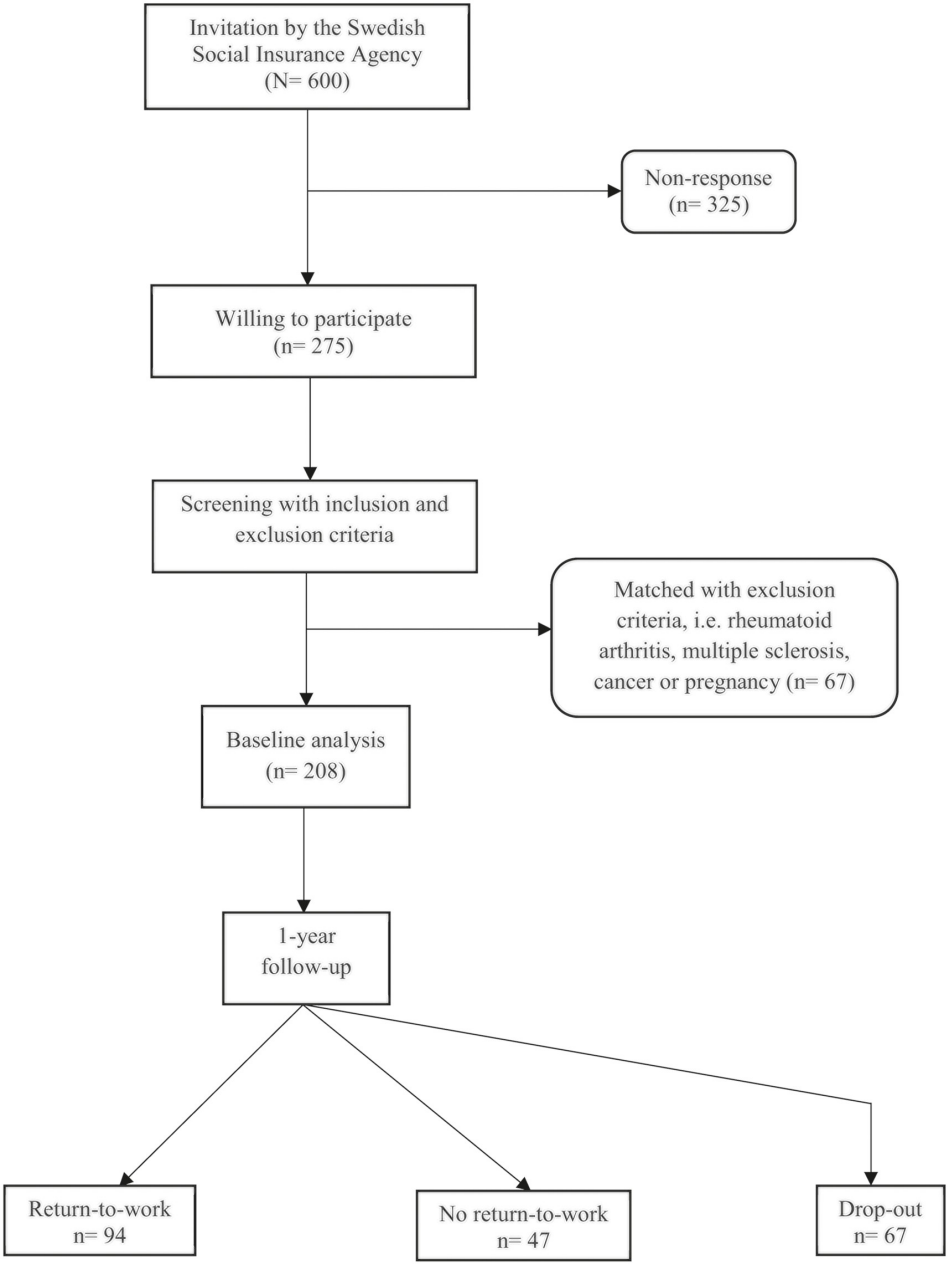
Flow chart of the study population at baseline and at follow-up.

Of the 600 women initially contacted, 275 responded to the survey. Sixty-seven were excluded based on the exclusion criteria, resulting in 208 women at baseline. After 1 year, a follow-up survey was sent to the baseline participants and 141/208 women responded, corresponding to a response rate of 68% (Fig 1). An attrition analysis on age, life-long pain duration, pain intensity, work ability, and well-being showed no significant differences in baseline values between the dropouts and those remaining at follow-up (S1 Table). There was no multi-collinearity between the predictors in the prediction model, as the variance inflation factor was ≤ 1.04 [56].

Table 1 represents the baseline characteristics of the 94 participants who had RTW and the 47 who had NRTW at the 1-year follow-up. The group who had RTW rated less pain intensity, more coping through increasing behavioral activities and more strongly believed in returning to work within 6 months. Conversely, the group who NRTW received more social support outside work. There were also significant differences between the groups concerning age, cohabitation, and economic situation.

**Table 1:** Baseline characteristics and study variables of participants who had returned to work (RTW) and who had not returned to work (NRTW) at the 1-year follow-up.

Baseline characteristics	RTW (n = 94)	NRTW (n = 47)	*p*-value
Age (M, range), years	49.04 (23–63)	53.51 (24–64)	**0.007**
Cohabitation, n (%)			**0.04**
Living with partner	75 (79.8)	30 (63.8)	
Living alone	17 (18.1)	12 (25.6)	
Living apart	2 (2.1)	5 (10.6)	
Children living at home, n (%)			0.37
No	53 (56.4)	29 (64.4)	
Yes	41 (43.6)	16 (35.6)	
Education, n (%)			0.69
Elementary	13 (13.8)	10 (21.3)	
Upper secondary	45 (47.9)	22 (46.8)	
University	32 (34.0)	14 (29.8)	
Others	4 (4.3)	1 (2.1)	
Economic situation, n (%)			**0.004**
Very dissatisfied	5 (5.8)	9 (19.2)	
Dissatisfied	19 (20.4)	8 (17.0)	
Acceptable	36 (38.5)	21 (44.7)	
Good	27 (28.7)	5 (10.6)	
Very good	6 (6.6)	4 (8.5)	
Years in the workforce (M, range)	30.04 (6–46)	32 (3–47)	0.25
Type of work^1^, n (%)			0.71
White-collar	35 (37.2)	16 (34.0)	
Blue-collar	59 (62.8)	31 (66.0)	
Stress in the last 6 months			**0.04**
All of the time	11 (11.8)	12 (26.7)	
Almost all of the time	24 (25.8)	16 (35.6)	
Some of the time	41 (44.1)	14 (31.1)	
A small part of the time	15 (16.1)	2 (4.4)	
Not at all	2 (2.2)	1 (2.2)	
Pain area, n (%)			0.52
Neck/shoulders	65 (69.1)	32 (68.1)	
Back	63 (67.0)	38 (80.9)	
Neck/shoulders and back	36 (25.5)	25 (17.7)	
Behavioral activity^2^ (M ± SD)	13.10 ± 4.9	11.13 ± 5.0	**0.03**
Ignore sensations^2^ (M ± SD)	13.29 ± 5.0	13.69 ± 6.4	0.69
Self-efficacy (M ± SD)	30.62 ± 4.4	29.26 ± 7.0	0.23
Sense of coherence (M ± SD)	63.65 ± 12.5	58.50 ± 13.16	**0.03**
Physical activity, n (%)			0.25
0 days/week	12 (12.8)	7 (15.0)	
1–3 days/week	48 (51.1)	16 (34.0)	
4–5 days/week	24 (25.5)	12 (25.5)	
6–7 days/week	10 (10.6)	12 (25.5)	
Beliefs about returning to work^3^ (M ± SD)	7.69 ± 3.2	4.24 ± 4.1	**< 0.001**
Pain intensity (M ± SD)	3.70 ± 1.2	4.76 ± 0.8	**< 0.001**
Social support outside work (M ± SD)	3.13 ± 0.8	3.58 ± 0.7	**0.001**
Depression (M ± SD)	5.46 ± 3.8	7.40 ± 5.0	**0.01**
Job strain (M ± SD)	0.77 ± 0.2	0.86 ± 0.2	**0.01**
Life-long pain duration (M, range), months	81.63 (3–420)	100.45 (4–360)	0.32

^1^Examples of white-collar work include office administration, nursing and teaching; example of blue-collar work include elderly care, childcare, and cleaning.

^2^Coping through increasing behavioral activities and coping by ignoring sensations were measured using the Coping Strategies Questionnaire, with scores ranging from 0 to 31 points and higher values indicating more frequent use of the coping strategy.

^3^Beliefs about returning to the same work within 6 months were assessed using a single question and rated on a 10-point Likert scale (1 = highly unlikely to return to the same work; 10 = highly likely to return to the same work), M mean; SD standard deviation.

**Fig 2:**
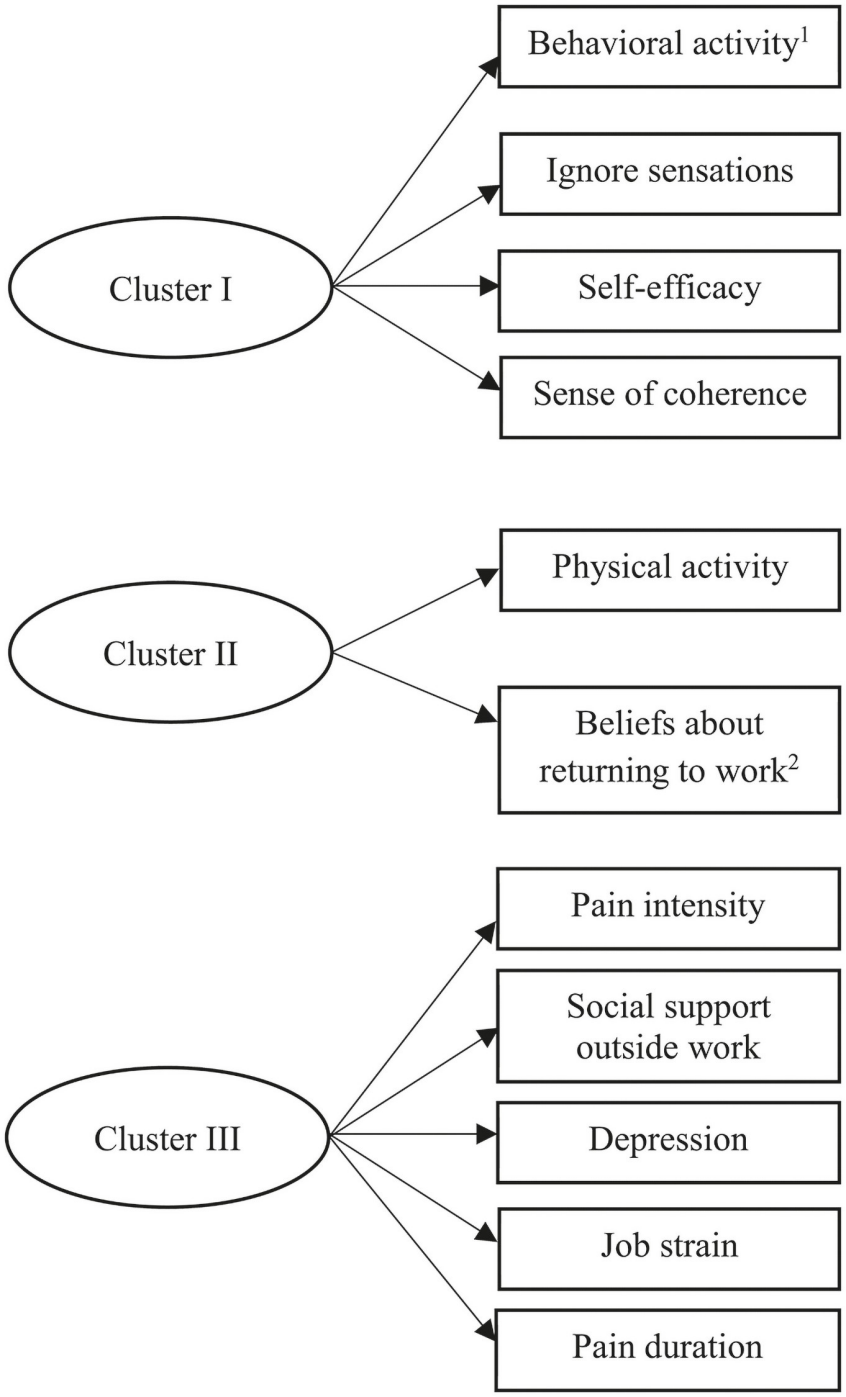
Illustrates the three clusters. ^1^Coping through increasing behavioral activities; ^2^Beliefs about returning to the same work within 6 months.

Fig 2 (based on the dendrogram in S1 Fig) shows the cluster analysis. Although four predictors could fit into the prediction model, we chose three (plus age as a covariate) because the difference in distance (i.e., squared Euclidean distance of z-scores) between clusters in the 3-cluster and the 4-cluster solution was small. Cluster I consisted of coping through increasing behavioral activities, ignore sensations, self-efficacy, and a sense of coherence. Increasing behavioral activities was chosen from the cluster because it had the highest relative dispersion in values. Moreover, the results of a previous study support this choice, because using behavioral activities as a coping strategy might help women deal with their pain [57]. Cluster II consisted of physical activity and beliefs about returning to the same work within 6 months. Both variables had the same dispersion in values, and so belief about returning to the same work within 6 months was selected on the basis of previous research showing that such beliefs are important for RTW [23]. Furthermore, earlier studies in this population found that beliefs about returning to the same work within 6 months was correlated with work ability [44], and that work ability predicted RTW [58]. Cluster III consisted of pain intensity, social support outside work, depression, job strain, and pain duration. Social support outside work was chosen from this cluster because it had the highest relative dispersion in values. According to previous studies, women on sick leave for MSP may need social support from immediate family, relatives and the surrounding environment to cope with pain and to assist in planning for RTW [30].

Table 2 shows the results of the multiple logistic regression analysis. All three predictors–coping through increasing behavioral activities, beliefs about returning to the same work within 6 months, and social support outside work–were significantly associated with RTW, and the results remained significant after controlling for age in the adjusted analysis. More specifically, women who more frequently used behavioral activities to cope with pain (OR: 1.14, 95% CI: 1.03–1.25) and more strongly believed they would return to the same work within 6 months (OR: 1.22, 95% CI: 1.10–1.37) had an increased probability of RTW at the 1-year follow-up. Women who had more social support outside work showed a decreased chance of RTW (OR: 0.50, 95% CI: 0.28–0.92). The regression model was statistically significant (*p* < 0.001), and 34% of the variance in the outcome variable was explained by the predictors.

**Table 2:** Multiple logistic regression analysis of the selected predictors at baseline and return to work at 1-year follow-up.

Predictors	Unadjusted analysis			Adjusted analysis^1^		
	SE	OR (95% CI)	*p*-value	SE	OR (95% CI)	*p*-value
Behavioral activity^2^	0.05	1.12 (1.02–1.22)	0.02	0.05	1.14 (1.03–1.25)	0.008
Beliefs about returning to work^3^	0.06	1.24 (1.12–1.38)	< 0.001	0.06	1.22 (1.10–1.37)	< 0.001
Social support outside work^4^	0.31	0.49 (0.26–0.90)	0.02	0.31	0.50 (0.28–0.92)	0.03
Age				0.03	0.94 (0.89–0.99)	0.04
*Overall model for the women*	*R*^*2*^ = 0.30, χ^2^ = 31.83, *p* < 0.001			*R*^*2*^ = 0.34, χ^2^ = 36.71, *p* < 0.001		

^1^Age was controlled for in the adjusted analysis;

^2^Coping through increasing behavioral activities, such as leisure activities, reading, and socialization was measured using the Coping Strategies Questionnaire, with scores ranging from 0 to 31 points and higher values indicating more frequent use of the coping strategy.

^3^Beliefs about returning to the same work within 6 months were assessed using a single question and rated on a 10-point Likert scale (1 = highly unlikely to return to the same work; 10 = highly likely to return to the same work).

^4^Social support outside work was measured using three items in the Multidimensional Pain Inventory, with scores ranging from 0 to 6 and higher values indicating higher social support.

SE standard error; OR Odds Ratio; CI Confidence Interval.

## Discussion

The present results show that women who more frequently used behavioral activities as coping strategies and who more strongly believed they would return to the same work within 6 months had an increased chance of RTW, whereas women with higher social support outside work were less likely to RTW.

Coping through increasing behavioral activities such as leisure activities, reading and socialization was positively associated with RTW. This is consistent with a previous study suggesting that coping strategies such as relaxation, stress management, and activity training helped women on sick leave for MSP increase their ability to control and decrease the pain, which may be an important and effective tool for early RTW [59]. Another study found that coping with daily activities outside work was related to RTW among people with long-term MSP [20]. It may be that the women in the present study who had RTW were able to work despite the pain because they were coping with pain by increasing these behavioral activities. Indeed, the RTW group rated their pain intensity as 3.7 on average (scale of 0–6) and their behavioral activities as 13.1 on average (scale of 0–31), while the NRTW group rated their pain intensity as 4.8 and behavioral activities as 11.1 (Table 1). Consequently, further study is warranted to see whether pain intensity is a mediator in the relationship between behavioral activities and RTW in this population.

We found an association between beliefs about returning to the same work within 6 months and RTW. Our finding is consistent with an earlier study among women on sick leave showing that a positive expectation/belief regarding RTW within a year was associated with the ability to work and volition that facilitated RTW [18]. In addition, negative recovery beliefs have been found to be a risk factor for NRTW (i.e., sustained long-term sick leave) among individuals with non-specific chronic MSP [60]. Similarly, a systematic review investigating belief in recovery and its relationship with RTW [33] showed that positive work-related recovery beliefs predicted RTW. Believing in RTW should perhaps be a goal in efforts to facilitate RTW among individuals on sick leave for long-term MSP [61, 62]. Such beliefs could be a target for change in rehabilitation focused on RTW for this group [19]. The reasoning here is that believing in RTW may change the individuals’ attitudes, because attitudes originate from beliefs, which may increase their use of coping strategies such as behavioral activities that can help them manage their pain, thus supporting them in their RTW process.

Our results also indicate that social support outside work negatively predicted RTW. This result is in contrast to previous findings regarding social support and RTW. A qualitative study among workers on sick leave suggested that social support either from work or outside work was a positive indicator for RTW, although the authors recommended using quantitative measures of social support to verify their findings [30]. A quantitative study, however, found that social support from a partner might not be related to RTW among female workers on sick leave [32]. Regardless of this discrepancy, an explanation for our result could be that when individuals receive ample support from family and friends, they tend to feel comfortable about not returning to work. It is possible that having a great deal of social support may cause people to believe they no longer have the capacity to manage things without someone’s help and this belief may exacerbate their pain-related fear. This may be the reason they avoid doing their daily activities [63], which could eventually impede their RTW. A reason for the contradiction between our results and previous findings could be that previous studies measured social support in general [30, 32], which covers all aspects in life as a whole, including the quality of the partner relationship, satisfaction, and social integration. However, in the present study, social support was assessed by pain-related social support items, covering pain-related support, concern, and consideration from family and friends [47]. In addition, social support from the workplace might be more relevant than social support from family in the RTW process.

The present findings are in agreement with results from previous studies on RTW among men and women with MSP, particularly for the predictors “coping through increasing behavioral activities” and “beliefs about returning to the same work within 6 months.” However, this was not the case for the predictor “social support outside work,” as our result was partly in agreement with previous findings among men and women with MSP. A quantitative study [31] investigating social support outside work (i.e., any type of support from family and friends) among men and women with musculoskeletal injury found that among women, receiving support from family was negatively associated with RTW whereas support from friends was positively associated with RTW; but no such association was observed among men [31]. Before giving a recommendation to investigate women separately in this regard, there is a need for further research which uses larger samples and considers different measurements of social support in this population.

### Strengths and limitations

The strengths of the present study were the prospective design that included a 1-year follow-up of 68% of the participants, the selection of participants based on ICD-10 codes provided by a physician, and the fact that the predictors were selected without capitalizing on the relationship observed between predictors and the outcome.

One limitation of the study is its relatively small sample size, which allowed only a limited number of predictors to be considered simultaneously. Because the participants were not randomly selected, there may be sampling bias in the study, which may affect the external validity. A non-response analysis could not be conducted because we had no access to non-respondents’ data, as the participants were invited by the Swedish Social Insurance Agency. It is unknown whether the participants had first returned to work and then relapsed to being on sick leave again before the follow-up measurement. It was also unknown whether the participants working < 50% at baseline had opportunities to receive support from the workplace. If such support was available, the participants who were working to some extent at baseline may have been more likely to RTW than the participants who did not work at all. Further, information was lacking on whether the participants received treatment, and whether they received or had been offered modified duties or support from supervisors/co-workers during the year between baseline and follow-up. Another limitation is that there may be common-method bias in the results because self-reported data were used to measure the predictors and outcome. Similarly, because data were measured subjectively, we were unable to ensure that the participants were only considering pain in the neck/shoulder and/or back when answering the questions. We had no information on the psychometric properties of the single-item tools used in the study (physical activity, belief about RTW within 6 months, and life-long pain duration). As the small sample size did not support more than two groups, the authors set the cut-off point of RTW at working > 50% of their service. This might have affected the external validity of the present study. Moreover, because all of the participants were women, the results cannot be generalized to men. Further studies should use larger samples to test expanded models of relevant predictors including potential mediating and/or moderating factors.

## Conclusions

The present study found that coping through increasing behavioral activities such as leisure activities, reading, and socialization as well as beliefs about returning to the same work within 6 months, increased the probability of RTW, whereas social support outside work decreased the chance of RTW at a 1-year follow-up among women with long-term neck/shoulder and/or back pain. The predictors highlighted here can be considered by healthcare professionals aiming to facilitate RTW in this population. Moreover, healthcare professionals should consider whether individuals are getting more social support outside work when supporting them in their RTW process.

## Supporting information

S1 Fig Illustrates dendrogram from cluster analysis. ^1^Behavioral activity = Coping through increasing behavioral activities; ^2^Beliefs at work = Beliefs about returning to the same work within 6 months.
(PDF)

S1 Table Difference in mean at baseline data between participants and dropouts at 1-year follow-up by age, pain duration, pain intensity, work ability, and well-being. (PDF)

S1 File (PDF)

S2 File (PDF)

## References

[1] Waddell G, Burton AK. Is work good for your health and well-being? London. UK: TSO (The Stationery Office); 2006.

[2] Hubertsson J, Englund M, Hallgårde U, Lidwall U, Löfvendahl S, Petersson IF. Sick leave patterns in common musculoskeletal disorders–a study of doctor prescribed sick leave. BMC Musculoskelet Disord. 2014;15:176. doi: 10.1186/1471-2474-15-176 24886568 PMC4060756

[3] Pekkala J, Rahkonen O, Pietiläinen O, Lahelma E, Blomgren J. Sickness absence due to different musculoskeletal diagnoses by occupational class: a register-based study among 1.2 million Finnish employees. Occup Environ Med. 2018:oemed-2017-104571. doi: 10.1136/oemed-2017-104571 29382694

[4] Wijnhoven HAH, de Vet HCW, Picavet HSJ. Prevalence of musculoskeletal disorders is systematically higher in women than in men. Clin J Pain. 2006 October;22:717–724. doi: 10.1097/01.ajp.0000210912.95664.53 16988568

[5] Thorsen SV, Friborg C, Lundstrøm B, Kausto J, Örnelius K, Sundell T, et al. Previous sickness presence among long-term sick-listed in Norway and Sweden: A retrospective study of prevalence and self-reported reasons. Int J Soc Welf. 2015;24:376–387.

[6] Thorsen SV, Friborg C, Lundstrøm B, Kausto J, Örnelius K, Sundell T, et al. Sickness Absence in the Nordic Countries. Accessed: 20-Oct-2018. Nordic Social Statistics Committee (NOSOSCO), Copenhagen. ISBN 978-87-90248-67-3; 2015.

[7] The Swedish Social Insurance Agency. Social Insurance in Figures 2018. Sweden: The Swedish Social Insurance Agency. Accessed: 25-Aug-2018. SE–103 51 Stockholm. Report No. ISBN: 978-91-7500-404-4; 2018.

[8] Vingård E, Alexanderson K, Norlund A. Chapter 9. Consequences of being on sick leave. Scand J Public Heal Suppl. 2004;32:207–215. doi: 10.1080/14034950410021899 15513658

[9] Ockander M, Timpka T. A female lay perspective on the establishment of long-term sickness absence. Int J Soc Welf. 2001;10:74–79.

[10] Ockander MK, Timpka T. Women’s experiences of long term sickness absence: Implications for rehabilitation practice and theory. Scand J Public Health. 2003;31:143–148. doi: 10.1080/14034940210134095 12745765

[11] Gustafsson K, Marklund S. Consequences of sickness presence and sickness absence on health and work ability: A Swedish prospective cohort study. Int J Occup Med Environ Health. 2011;24:153–165. doi: 10.2478/s13382-011-0013-3 21526385

[12] Henschke N, Kamper SJ, Maher CG. The epidemiology and economic consequences of pain. Mayo Clin Proc. 2015;90:139–147. doi: 10.1016/j.mayocp.2014.09.010 25572198

[13] Jan de Kok, Vroonhof P, Snijders J, Roullis G, Clarke M, Peereboom K, et al. Work-related musculoskeletal disorders: prevalence, costs and demographics in the EU. ISBN: 978-92-9479-145-0; 2019.

[14] Steenstra IA, Lee H, De Vroome EMM, Busse JW, Hogg-Johnson SJ. Comparing current definitions of return to work: A measurement approach. J Occup Rehabil. 2012;22:394–400. doi: 10.1007/s10926-011-9349-6 22415602

[15] Wasiak R, Young AE, Roessler RT, McPherson KM, Van Poppel MNM, Anema JR. Measuring return to work. J Occup Rehabil. 2007;17:766–781. doi: 10.1007/s10926-007-9101-4 17929149

[16] Josephson M, Heijbel B, Voss M, Alfredsson L, Vingård E. Influence of self-reported work conditions and health on full, partial and no return to work after long-term sickness absence. Scand J Work Environ Heal. 2008;34:430–437. doi: 10.5271/sjweh.1289 19137204

[17] Ahlstrom L, Dellve L, Hagberg M, Ahlberg K. Women with Neck Pain on Long-Term Sick Leave—Approaches Used in the Return to Work Process: A Qualitative Study. J Occup Rehabil. 2017;27:92–105. doi: 10.1007/s10926-016-9636-3 26944045 PMC5306228

[18] Claréus B, Renström EA. Patients’ return-to-work expectancy relates to their beliefs about their physician’ s opinion regarding return to work volition and ability. J Pain Res. 2019;12:353–362. doi: 10.2147/JPR.S179061 30697062 PMC6339447

[19] Brendbekken R, Vaktskjold A, Harris A, Tangen T. Predictors of return-to-work in patients with chronic musculoskeletal pain: A randomized clinical trial. J Rehabil Med. 2018;50:193–199. doi: 10.2340/16501977-2296 29206274

[20] Sviridova O, Gard G, Michaelson P. Predictors for return to work after multimodal rehabilitation in persons with persistent musculoskeletal pain. Edorium J Disabil Rehabil. 2018;4.

[21] Iles RA, Davidson M, Taylor NF. Psychosocial predictors of failure to return to work in non-chronic non-specific low back pain: A systematic review. Occup Environ Med. 2008;65:507–517. doi: 10.1136/oem.2007.036046 18417552

[22] Heymans MW, De Vet HCW, Knol DL, Bongers PM, Koes BW, Van Mechelen W. Workers’ beliefs and expectations affect return to work over 12 months. J Occup Rehabil. 2006;16:685–695. doi: 10.1007/s10926-006-9058-8 17063403

[23] Steenstra IA, Munhall C, Irvin E, Oranye N, Passmore S, Van Eerd D, et al. Systematic Review of Prognostic Factors for Return to Work in Workers with Sub Acute and Chronic Low Back Pain. J Occup Rehabil. 2017;27:369–381. doi: 10.1007/s10926-016-9666-x 27647141 PMC5591348

[24] Hansen A, Edlund C, Henningsson M. Factors relevant to a return to work: A multivariate approach. Work. 2006;26:179–190. 16477110

[25] Holmgren K, Ivanoff SD. Women on sickness absence—Views of possibilities and obstacles for returning to work. A focus group study. Disabil Rehabil. 2004;26:213–222. doi: 10.1080/09638280310001644898 15164955

[26] Rashid M, Heiden M, Nilsson A, Kristofferzon M-L. Do work ability and life satisfaction matter for return to work? Predictive ability of the work ability index and life satisfaction questionnaire among women with long-term musculoskeletal pain. BMC Public Health. 2021;21:584. doi: 10.1186/s12889-021-10510-8 33761910 PMC7992335

[27] Ahlberg-Hulten GK, Theorell T, Sigala F. Social support, job strain and musculoskeletal pain among female health care personnel. Scand J Work Environ Health. 1995;21:435–439. doi: 10.5271/sjweh.59 8824749

[28] Grant M, Rees S, Underwood M, Froud R. Obstacles to returning to work with chronic pain: In-depth interviews with people who are off work due to chronic pain and employers. BMC Musculoskelet Disord. 2019;20:1–15. 31656184 10.1186/s12891-019-2877-5 PMC6815386

[29] Englund ACD, Rydström I, Dellve L, Ahlstrom L. Social support outside work and return to work among women on long-term sick leave working within human service organizations. Appl Nurs Res. 2016;30:187–193. doi: 10.1016/j.apnr.2015.06.007 27091277

[30] Lysaght RM, Larmour-Trode S. An exploration of social support as a factor in the return-to-work process. Work. 2008;30:255–266. 18525149

[31] Prang K, Berecki-gisolf J, Newnam S. Recovery from musculoskeletal injury: the role of social support following a transport accident. Health Qual Life Outcomes. 2015:1–17. 26138816 10.1186/s12955-015-0291-8 PMC4490643

[32] Dellve L, Ahlborg T. Partner relationships and long-term sick leave among female workers: Consequences and impact on dimensions of health and return to work. Scand J Caring Sci. 2012;26:720–729. doi: 10.1111/j.1471-6712.2012.00985.x 22515840

[33] Rashid M, Kristofferzon M, Nilsson A, Heiden M. Factors associated with return to work among people on work absence due to long-term neck or back pain: a narrative systematic review. BMJ Open. 2017;7:1–10. doi: 10.1136/bmjopen-2016-014939 28674139 PMC5734441

[34] Lewis C, Mathiassen SE. State of Knowledge Report: Physical work, gender, and health in working life. Swedish work environment authority. Accessed: 15-Jan-2017. ISSN 1650-3171. Report 2013:9; 2013.

[35] Svanholm F, Liedberg GM, Löfgren M, Björk M. Factors of importance for return to work, experienced by patients with chronic pain that have completed a multimodal rehabilitation program–a focus group study. Disabil Rehabil. 2020;0:1–9. doi: 10.1080/09638288.2020.1780479 32580604

[36] Björklund M, Djupsjöbacka M, Svedmark Å, Häger C. Effects of tailored neck-shoulder pain treatment based on a decision model guided by clinical assessments and standardized functional tests. A study protocol of a randomized controlled trial. BMC Musculoskelet Disord. 2012;13. doi: 10.1186/1471-2474-13-75 22607546 PMC3517365

[37] Nazarov S, Manuwald U, Leonardi M, Silvaggi F, Foucaud J, Lamore K, et al. Chronic diseases and employment: Which interventions support the maintenance of work and return to work among workers with chronic illnesses? a systematic review. Int J Environ Res Public Health. 2019;16:1–14. 10.3390/ijerph16101864 PMC6572561 31137817

[38] Margolis RB, Tait RC, Krause SJ. A rating system for use with patient pain drawings. Pain. 1986 January;24:57–65. doi: 10.1016/0304-3959(86)90026-6 2937007

[39] Lydell M, Grahn B, Månsson J, Baigi A, Marklund B. Predictive factors of sustained return to work for persons with musculoskeletal disorders who participated in rehabilitation. Work. 2009;33:317–328. doi: 10.3233/WOR-2009-0879 19759430

[40] Jensen IB, Linton SJ. Coping Strategies Questionnaire (CSQ): Reliability of the Swedish version of the CSQ. Scand J Behav Ther. 1993;22:139–145.

[41] Ferreira-Valente MA, Pais Ribeiro JL, Jensen MP, Almeida R. Coping with Chronic Musculoskeletal Pain in Portugal and in the United States: A Cross-Cultural Study. Pain Med. 2011;12:1470–1480. doi: 10.1111/j.1526-4637.2011.01208.x 21834916

[42] Lau OWY, Leung LNY, Wong LOL. Cognitive behavioural techniques for changing the coping skills of patients with chronic pain. Hong Kong J Occup Ther. 2002;12:13–20.

[43] Quartana PJ, Campbell CM, Edwards RR. Pain catastrophizing a critical review. Expert Rev Neurother. 2009;9:745–758. doi: 10.1586/ern.09.34 19402782 PMC2696024

[44] Rashid M, Kristofferzon M-L, Heiden M, Nilsson A. Factors related to work ability and well-being among women on sick leave due to long-term pain in the neck/shoulders and/or back: A cross-sectional study. BMC Public Health. 2018;18:1–8. doi: 10.1186/s12889-018-5580-9 29848306 PMC5977487

[45] Luszczynska A, Scholz U, Schwarzer R. The General Self-Efficacy Scale: Multicultural Validation Studies. J Psychol. 2005;139:439–547. doi: 10.3200/JRLP.139.5.439-457 16285214

[46] Antonovsky A. The structure and properties of the sense of coherence scale. Soc Sci Med. 1993 March;36:725–733. doi: 10.1016/0277-9536(93)90033-z 8480217

[47] Bergström G, Jensen IB, Bodin L, Linton SJ, Nygren ÅL, Carlsson SG. Reliability and factor structure of the multidimensional pain inventory—Swedish language version (MPI-S). Pain. 1998;75:101–110. doi: 10.1016/S0304-3959(97)00210-8 9539679

[48] Zigmond AS, Snaith RP. The Hospital Anxiety and Depression Scale. Acta Psychiatr Scand. 1983;67:361–370. doi: 10.1111/j.1600-0447.1983.tb09716.x 6880820

[49] Albert PR. Why is depression more prevalent in women? J Psychiatry Neurosci. 2015;40:219–221. doi: 10.1503/jpn.150205 26107348 PMC4478054

[50] Sanne B, Torp S, Mykletun A, Dahl A a. The Swedish Demand-Control-Support Questionnaire (DCSQ): factor structure, item analyses, and internal consistency in a large population. Scand J Public Health. 2005;33:166–174. doi: 10.1080/14034940410019217 16040456

[51] Chungkham HS, Ingre M, Karasek R, Westerlund H, Theorell T. Factor Structure and Longitudinal Measurement Invariance of the Demand Control Support Model: An Evidence from the Swedish Longitudinal Occupational Survey of Health (SLOSH). PLoS One. 2013;8. 10.1371/journal.pone.0070541 PMC3741382 23950957

[52] Peduzzi P, Concato J, Kemper E, Holford TR, Feinstem AR. A simulation study of the number of events per variable in logistic regression analysis. J Clin Epidemiol. 1996;49:1373–1379. doi: 10.1016/s0895-4356(96)00236-3 8970487

[53] Heiden M, Mathiassen SE, Garza J, Liv P, Wahlström J. A Comparison of Two Strategies for Building an Exposure Prediction Model. Ann Occup Hyg. 2015;60:74–89. doi: 10.1093/annhyg/mev072 26424806

[54] Harrell FE. Regression Modeling Strategies: with Applications to Linear Models, Logistic Regression, and Survival Analysis. Springer New York; 2010.

[55] Sanche R, Lonergan K. Variable Reduction for Predictive Modeling with Clustering. Casualty Actuar Soc Forum. 2006:89–100.

[56] O’Brien RM. A caution regarding rules of thumb for variance inflation factors. Qual Quant. 2007;41:673–690.

[57] Brendbekken R, Harris A, Ursin H, Eriksen HR, Tangen T. Multidisciplinary Intervention in Patients with Musculoskeletal Pain: a Randomized Clinical Trial. Int J Behav Med. 2016;23:1–11. doi: 10.1007/s12529-015-9486-y 25920707 PMC4747998

[58] Rashid M, Heiden M. Do work ability and well-being matter for return to work? Cut-off points for the Work Ability Index and Life Satisfaction Questionnaire among women with long-term musculoskeletal pain. 2021:1–14. doi: 10.1186/s12889-021-10510-8 33761910 PMC7992335

[59] Marhold C, Linton SJ, Melin L. A cognitive-behavioral return-to-work program: effects on pain patients with a history of long-term versus short-term sick leave. Pain. 2001 March;91:155–163. doi: 10.1016/s0304-3959(00)00431-0 11240088

[60] Busch H, Göransson S, Melin B. Self-efficacy beliefs predict sustained long-term sick absenteeism in individuals with chronic musculoskeletal pain. Pain Pract. 2007;7:234–240. doi: 10.1111/j.1533-2500.2007.00134.x 17714102

[61] Siegert RJ, Taylor WJ. Theoretical aspects of goal-setting and motivation in rehabilitation. Disabil Rehabil. 2004;26:1–8. doi: 10.1080/09638280410001644932 14660192

[62] Lydell M, Hildingh C, Månsson J, Marklund B, Grahn B. Thoughts and feelings of future working life as a predictor of return to work: A combined qualitative and quantitative study of sick-listed persons with musculoskeletal disorders. Disabil Rehabil. 2011;33:1262–1271. doi: 10.3109/09638288.2010.527029 21034307

[63] Gatchel RJ, Neblett R, Kishino N, Ray CT. Fear-Avoidance Beliefs and Chronic Pain. J Orthop Sport Phys Ther. 2016;46:38–43. doi: 10.2519/jospt.2016.0601 26828236

