# Supplementary material for: Predictors of return to work among women with long-term neck/shoulder and/or back pain: A 1-year prospective study
Source: PLoS One. 2021 Nov 23;16(11):e0260490. doi: 10.1371/journal.pone.0260490 (PMC8610267; doi:10.1371/journal.pone.0260490)
Supplement: S1 Fig — 1Behavioral activity = Coping through increasing behavioral activities; 2Beliefs at work = Beliefs about returning to the same work within 6 months. (PDF) [file pone.0260490.s001.pdf]

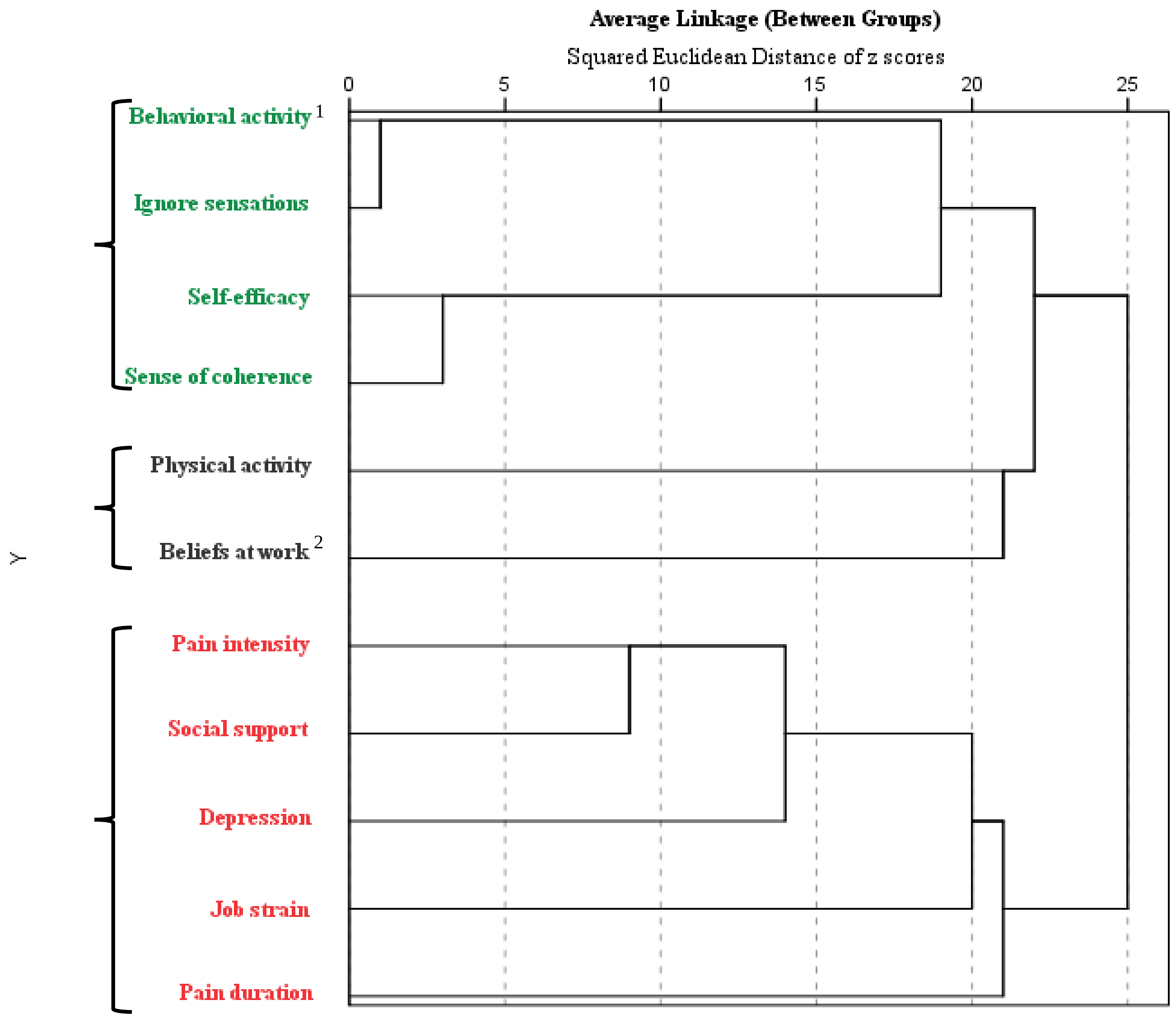

S1 Fig. Illustrates dendrogram from cluster analysis. [1]Behavioral activity= Coping through increasing behavioral activities; [2]Beliefs at work= Beliefs about returning to the same work within 6 months.
