# Supplementary material for: Predictors of return to work among women with long-term neck/shoulder and/or back pain: A 1-year prospective study
Source: PLoS One. 2021 Nov 23;16(11):e0260490. doi: 10.1371/journal.pone.0260490 (PMC8610267; doi:10.1371/journal.pone.0260490)
Supplement: S1 Table — (PDF) [file pone.0260490.s002.pdf]

S1 Table. Difference in mean at baseline data between participants and dropouts at 1-year follow-up by age, pain duration, pain intensity, work ability, and well-being.

**Variables**	**Follow-up group (n= 141) (M±SD)**	**Dropout group (n= 67) (M±SD)**	***t*-test**	***p*-value**
Age	50.53±9.3	47.73±10.2	1.89	0.06
Pain duration[1]	87.49±101.1	69.00±94.1	1.22	0.23
Pain intensity	4.05±1.2	4.36±1.1	-1.84	0.07
Work ability	23.20±7.8	22.43±7.7	0.67	0.50
Well-being	45.30±9.5	46.28±9.7	-0.62	0.53

Life-long pain duration in month, M Mean, SD Standard Deviation
