# Supplementary material for: Predictors of return to work among women with long-term neck/shoulder and/or back pain: A 1-year prospective study
Source: PLoS One. 2021 Nov 23;16(11):e0260490. doi: 10.1371/journal.pone.0260490 (PMC8610267; doi:10.1371/journal.pone.0260490)
Supplement: S1 File — (PDF) [file pone.0260490.s003.pdf]

ETIKPRÖVNINGSNÄMNDEN
EPN
REGIONALA · UPPSALA ·

BESLUT
2015-11-11 **Dnr 2015/424**

SÖKANDE FORSKNINGSHUVUDMAN
Högskolan i Gävle
801 76 Gävle

Forskare som genomför projektet:
Marja-Leena Kristofferzon
Akademin för Hälsa och Arbetsliv
Avd. för hälso- och vårdvetenskap
Högskolan i Gävle
801 76 Gävle

UPPGIFTER OM FORSKNINGSPROJEKTET ENLIGT ANSÖKAN INKOMMEN TILL NÄMNDEN 2015-10-19

Projektbeskrivning:
Kvinnor i arbetsför ålder med långvarig smärta: coping, coping-resurser, välbefinnande, arbetsförmåga och återgång i arbetet

---

Regionala etikprövningsnämnden i Uppsala meddelar följande

BESLUT

Nämnden bifaller ansökningen och godkänner med stöd av 6 § lagen (2003:460) om etikprövning av forskning som avser människor den forskning som anges i ansökan med följande villkor:

Etikprövningsnämnden godkänner delstudie 1-3 med följande villkor:

I informationsbladen till forskningspersonerna ska:

1. Formuleringen "ingen utomstående kan" bytas mot "ingen obehörig får" då det ytterst är en domstol som kan bestämma om vilka som får tillgång till grundmaterialet.

2. Det inte finnas någon logotype för Försäkringskassan då de inte är forskningshuvudman.

3. Marja-Leena Kristofferzon skrivas ut som enda huvudansvarig forskare och som sådan sättas först bland kontaktpersoner.

2015-11-11 **Dnr 2015/424**

Samtyckesblanketten skrivs på ett separat blad så att forskningspersonen kan ta övrig information med sig hem. I samtyckesblanketten ska det anges ett namn på det projekt samtycket avser.

Nämnden gör den sökande också uppmärksam på att det för delstudie 4 kan krävas en ny ansökan då nämnden inte har någon ansökningskategori som motsvarar vad som i ansökan betecknas som "tilläggsansökan".

Erinran

Godkännandet upphör att gälla om forskningen inte har påbörjats inom två år efter slutgiltigt beslut.

BESLUTET FÅR ÖVERKLAGAS
Se bifogad anvisning.

På nämndens vägnar

Johan Modin
Ordförande

Beslutande:
Johan Modin, rådman, ordförande
Beslutande: Ledamöter med vetenskaplig kompetens
Staffan Hygge miljöpsykologi (vetenskaplig sekreterare), Bo Lewin sociologi (vetenskaplig sekreterare), Lena Almqvist psykologi (föredragande), Daniela Andrén nationalekonomi, Henry Cöster teologi, Katarina Elofsson nationalekonomi, Kristina Haglund vårdvetenskap, David O. Kronlid etik och didaktik, Greta Ågren etologi
Ledamöter som företräder allmänna intressen
Sture Beckman, Barbro Larsson, Lina Nordquist, Michael Williams

Exp. till:
Forskare: Marja-Leena Kristofferzon
Forskningshuvudmannens företrädare: Akademichef Nader Ahmadi

Adress	Telefon	Fax	E-post
Box 1964<br>751 49 Uppsala	018-4717400	018-4717410	registrator@uppsala.epn.se

Dnr 2015/424

Hur man överklagar etikprövningsnämndens beslut

Vem får överklaga?

Överklagandet ska göras av **forskningshuvudmannen/ behörig företrädare.** Behörig företrädare får lämna skriftlig fullmakt till forskare som genomför projektet.

Var ska beslutet överklagas?

Nämndens beslut kan överklagas hos Centrala etikprövningsnämnden, Stockholm. Överklagandet ska dock skickas eller lämnas till: Regionala etikprövningsnämnden i Uppsala, Box 1964, 751 49 UPPSALA.

Har överklagandet inkommit i rätt tid överlämnar nämnden överklagandet och handlingarna till Centrala etikprövningsnämnden.

När ska beslutet senast överklagas?

Överklagandet ska ha kommit in till nämnden **inom tre veckor** från den dag Ni fick del av beslutet.

Vad ska överklagandet innehålla?

Överklagandet ska vara skriftligt och det ska vara undertecknat.

I skrivelsen ska Ni ange

- Ert namn, adress, personnummer/organisationsnummer och telefonnummer,

- vilket beslut som Ni överklagar t.ex. genom att ange beslutsdatum och ärendets diarienummer,

- hur Ni anser att nämndens beslut ska ändras och varför det ska ändras.

- eventuell fullmakt som bilaga.

Adress	Telefon	Fax	E-post
Box 1964	018-4717400	018-4717410	registrator@uppsala.epn.se
751 49 Uppsala			
