# Supplementary material for: Predictors of return to work among women with long-term neck/shoulder and/or back pain: A 1-year prospective study
Source: PLoS One. 2021 Nov 23;16(11):e0260490. doi: 10.1371/journal.pone.0260490 (PMC8610267; doi:10.1371/journal.pone.0260490)
Supplement: S2 File — (PDF) [file pone.0260490.s004.pdf]

HÖGSKOLAN
I GÄVLE
Akademin för hälsa och arbetsliv

Kvinnor i arbetsför ålder med långvarig smärta: hantering och resurser (coping), välbefinnande, arbetsförmåga och återgång i arbete

Du som har erfarenhet av att leva med långvarig smärta ifrån nacke, axlar, skuldror, eller rygg tillfrågas härmed om att delta i ett forskningsprojekt. Projektet är ett samarbete mellan Försäkringskassan i Region Gävleborg och Högskolan i Gävle. Syftet är att undersöka vilka hälsofrämjande faktorer och riskfaktorer som har betydelse för välbefinnande, arbetsförmåga och återgång i arbete hos kvinnor med långvarig smärta som är i arbete respektive sjukskrivna.

Du är en av 130 personer som får detta brev då du är sjukskriven för långvarig smärta. Att delta i studien innebär att du vid två tillfällen fyller i ett frågeformulär med ett års mellanrum. Tiden det tar att besvara frågorna beräknas till 45 minuter. Vid det första tillfället skickar Försäkringskassan i Region Gävleborg ut frågeformuläret eftersom endast de har dina uppgifter. Om du samtycker till att delta i studien, skickas frågeformuläret vid andra tillfället ut via Högskolan i Gävle. All information (personuppgifter*) som samlas in vid de två tillfällena kommer att behandlas så att ingen utomstående har tillgång till den. Resultatet från deltagarna kommer att jämföras med de deltagare som är i arbete. Deltagandet är frivilligt och du kan när som helst avbryta det utan närmare motivering och utan att handläggningen av ditt ärende på Försäkringskassan påverkas.

I det övre högra hörnet på frågeformuläret finns ett kodnummer. Om du inte besvarat frågeformuläret kommer du att få upp till två påminnelser om att göra det med ca två veckors mellanrum. Redovisningen av resultatet kommer att ske på gruppnivå så att ingen enskild deltagare kan identifieras. Din information kommer att förvaras inlåst under 10 år efter publicering och därefter förstöras.

Ansvarig för den information som du lämnar i frågeformuläret är Högskolan i Gävle som också är forskningshuvudman. Hanteringen av informationen följer Personuppgiftslagen (PUL 1998:204). Nedanstående personer är ansvariga för projektet och kan kontaktas om du önskar tillägga eller ändra något i dina svar. Enligt PUL har du rätt att en gång per år kostnadsfritt ta del av samtliga uppgifter som finns registrerade om dig och vid behov få eventuella fel rättade.

*Med personuppgifter avses de uppgifter som samlats in om dig via frågeformuläret. Dessa identifieras endast med ett kodnummer, inte med namn. Kodlistan som knyter kodnumret till namn förvaras inlåst vid Högskolan i Gävle.

HÖGSKOLAN
I GÄVLE
Akademin för hälsa och arbetsliv

Huvudansvariga forskare:

Marja-Leena Kristofferzon
Docent, Universitetslektor, Projektledare
Högskolan i Gävle
Avdelningen för hälso- och vårdvetenskap
e-post: mko@hig.se
Telefon: 026-648242

Annika Nilsson
Fil.dr, Universitetslektor
Högskolan i Gävle
Avdelningen för hälso- och vårdvetenskap
e-post: ans@hig.se
Telefon: 026-648282

Marina Heiden
Med.dr, Universitetslektor
Högskolan i Gävle
Avdelningen för arbets- och folkhälsovetenskap
e-post: marina.heiden@hig.se
Telefon: 026-648544

Mamunur Rashid
Doktorand
Högskolan i Gävle
Avdelningen för arbets- och folkhälsovetenskap
e-post: Mamunur.Rashid@hig.se
Telefon: 026-648110

HÖGSKOLAN
I GÄVLE
Akademin för hälsa och arbetsliv

Genom min underskrift samtycker jag till att delta i studien och att mina personuppgifter behandlas som beskrivits.

Behåll ett exemplar av ditt samtycke till att delta i studien. Det andra exemplaret av samtycket ska skickas tillbaka tillsammans med frågeformuläret.

…………………………………………………………………..
Ort och datum

……………………………………………………………………
Namn

……………………………………………………………………
Namnförtydligande

…………………………………………………………………….
Adress

…………………………………………………………………….
Telefonnummer

HÖGSKOLAN
I GÄVLE
Akademin för hälsa och arbetsliv

För att få ta del av dina erfarenheter av att leva med långvarig smärta kommer vi att vid ett senare tillfälle utföra intervjuer. Vi önskar därför kontakta ett antal deltagare som besvarat frågeformuläret för intervju. Kryssa i rutan och skicka tillbaka blanketten om du vill bli kontaktad för en sådan intervjustudie.

Ja, jag vill bli kontaktad för en intervjustudie. ☐

……………………………………………………………..
Ort och datum

………………………………………………………………
Namn

………………………………………………………………
Namnförtydligande
