# Supplementary material for: Predictors of return to work among women with long-term neck/shoulder and/or back pain: A 1-year prospective study
Source: PLoS One. 2021 Nov 23;16(11):e0260490. doi: 10.1371/journal.pone.0260490 (PMC8610267; doi:10.1371/journal.pone.0260490)
Supplement: Attachment — Submitted filename: Response to Reviewers.docx [file pone.0260490.s005.docx]

**Authors’ response to reviews**

**Title: Predictors of return to work among women with long-term neck/shoulder and/or back pain: a 1-year prospective study**

We thank the reviewer for his/her helpful comments and feel that the changes have improved the manuscript. We have now carefully considered and revised the manuscript based on the comments that were sent to us. The following is our point-by-point reply.

| **Editor’s Comments:** |  |
| --- | --- |
| Special attention should focus points 3, 4 and 5 raised by the referee due to their relevance. Accordingly, explain the difference between items chosen/listed by the pain severity MPI scale to infer pain intensity. Concerning the subscale selection from each questionnaire, why were pain catastrophizing (CSQ) and anxiety (HADS) not included since these are very relevant variables in pain experience? Concerning the rationale of the cluster analysis on all candidate predictors and then selection of one from each cluster, this must be better explained and justified. Specifically, the variables grouped under each cluster are in some cases very different: in cluster 3 pain intensity and social support do not assess similar constructs. In clinical terms this is highly speculative. | The points raised by the editor have been answered accordingly in reviewer’s comments 3, 4 and 5 in Methods section. |
|  |  |
| **Reviewer’s comments** |  |
| *General comments:* |  |
| 1. Why did the authors opt to use the term "long-term pain" and not "chronic pain"? If the type of pain was labeled as chronic it would be clear that only patients with pain over 3 months were included (as per the IASP definition) and this could help clarify the sample in the study. For example, it is not clear in the abstract the pain duration considered for inclusion. | Thank you for providing the insights. Chronic pain usually refers to persistent pain that is mainly used in a clinical context. The reason we used the term “long-term pain,” is because the participants in the study experienced pain for ≥ 3 months, but that was not persistent. We added text in the Abstract (page 2, lines 59–61). |
| 2. Globally, the paper is well written but would benefit from a revision for increased clarity in some sections. | Thank you. |
| *Abstract:* |  |
| 1. I suggest adding information on where patients were recruited (social security agency). | The information has been added (page 2, lines 59–60). |
| 2. In the Methods, it should be clear that clusters were used to identify one predictor from each "category". | This has now been clarified on page 2, lines 63–64. |
|  |  |
| INTRODUCTION |  |
| Page 4, line 121: "Increased work ability per se was found to be an important predictor for women with pain in the neck/shoulder and/or back" - It is not clear what work ability was a predictor of. Return to work? Or having pain? | Thank you for pointing this out. The change has been made accordingly (page 4, line 122). |
| Page 4, line 125: Please revise this sentence since it is a bit confusing. | The sentence has been rephrased (page 4, line 126). |
| METHODS |  |
| *Major issues:* |  |
| 1. Did the authors control for insurance or litigation issues among the participants? In case of sick leave due to work-related injuries this is usually a relevant factor associated with RTW. | This is an important question, raised by the reviewer, that insurance is a relevant factor related to RTW. However, the insurance system in Sweden is more centralized, meaning that if someone works in Sweden, they officially get connected to the Swedish Social Insurance Agency (SIA), and receive sick leave benefits due to diseases/disorders. And the participants’ information in this study was collected from the SIA. Because all the participants in this study had insurance, we did not control for the insurance. |
| 2. Is the cut-off for working or not working (50% from extent of employment) commonly used in these types of study or was it determined by the authors? Can some references be provided? It seems to me that, for example, it would not be appropriate to consider as RTW someone who was working 40% at baseline and 60% at follow-up. | The cut-off for RTW and NRTW based on 50% from the extent of employment was determined by the authors. There is no consensus on how to use the concept RTW. In the introduction, we described it with references – “The measures used vary with the population studied as well as across societies and countries, which have different healthcare systems and workers compensation regulations” (page 3, lines 106–108). |
| 3. The authors used the 3 original items from the pain severity MPI scale to infer pain intensity. However, this scale only has 2 items (suffering item was removed) according to the Swedish validation that the authors cite. What is the rationale to use 3 items? Providing a measure of reliability could provide a better sense of the adequacy of this scale, and I believe this data should be presented here, despite also being reported elsewhere.    Also, it’s not clear which MPI subscale was used to assess "social support outside work". I suggest that the authors mention the name of the original subscales and provide reliability measures for all questionnaires used. | The reviewer has raised an important question. Although the authors in Bergström, et al. (1998) [1] used 2 items in the scale, we cited the reference because they used the Swedish version of MPI first time in a Swedish context. However, we have no better explanation than the rationale behind using 3 items in this study was that we used 3 items in a previous study with the same sample, which was found good reliability. This has been mentioned with reference on page 7, line 218.    1. Bergström G, Jensen IB, Bodin L, Linton SJ, Nygren ÅL, Carlsson SG. Reliability and factor structure of the multidimensional pain inventory - Swedish language version (MPI-S). Pain. 1998;75:101–110.  Social support outside work was measured using the sub-scale social support from the MPI, which consists of three items. This has now been mentioned with the reliability of the scale on page 7, lines 220–226. |
| 4. Concerning the subscale selection from each questionnaire, why were pain catastrophizing (CSQ) and anxiety (HADS) not included? These are very relevant variables in pain experience. | Thank you for the suggestion. Considering reviewer’s comment, we did an exchange of ignore sensations with pain catastrophizing in a cluster analysis. The results from the analysis showed that pain catastrophizing was included in cluster I with variables – pain intensity, social support outside work, job strain, depression, behavioral activity, and physical activity. Cluster II contains three variables – self-efficacy, sense of coherence, and beliefs about RTW within 6 months. Cluster III consists of pain duration only. Furthermore, we found a significant difference between groups for pain catastrophizing. However, when we performed a logistic regression analysis considering life-long pain duration, beliefs about RTW within 6 months, and pain catastrophizing, only beliefs about RTW within 6 months was found to be significant in both unadjusted and adjusted (age). Therefore, we decided not to consider pain catastrophizing in the analysis.  There were mainly two reasons we chose depression. First, depression is more prevalent in women than in men [1]. Moreover, We found a negative correlation between depression and well-being in this population in a previous study, but that was not the case for anxiety [2].  1. Albert P. R. (2015). Why is depression more prevalent in women? Journal of psychiatry & neuroscience: JPN, 40(4), 219–221.  2. Rashid M, Kristofferzon M-L, Heiden M, Nilsson A. Factors related to work ability and well-being among women on sick leave due to long-term pain in the neck/shoulders and/or back: A cross-sectional study. BMC Public Health. 2018;18:1–8. |
| 5. The authors perform a cluster analysis on all candidate predictors and then select one from each cluster, on the basis that they would provide similar information. However, I think that the rationale behind this decision must be better explained and justified. Though some variables may assess similar constructs, they are nonetheless different. In fact, the variables grouped under each cluster are in some cases very different: for example in cluster 3 pain intensity and social support do not assess similar constructs. Using this approach, relevant information may have been excluded from the analyses. For example, pain duration is very relevant to predict RTW.  The problem of multicollinearity can be addressed by analyzing the VIF values of the models. | We appreciate the reviewer’s comment. More information about the rationale behind choosing one predictor from each cluster has been added (page 11, lines 298–300, 302–303 and 310–312, respectively). We agree with the reviewer that pain duration could be a relevant predictor; but the predictor did not show statistically significant difference between the groups in this sample (see in Table 1).    Multicollinearity has now been checked to measure VIF between predictors in the model (page 9, line 284–285). |
| 6. What criteria were followed to determine the number of predictors in the model? The authors state in the limitations that the sample size precluded the inclusion of more variables, but a sample size of 141 would allow for more than 3 DVs. | We appreciate the reviewer’s comment. As you pointed out, we are able to test 4 predictors in the model according to our sample size (RTW= 94, and NRTW= 47) [1]. We used 3 predictors and one controlling variable (age) (see in Table 2).    1. Peduzzi P, Concato J, Kemper E, Holford TR, Feinstem AR. A simulation study of the number of events per variable in logistic regression analysis. J Clin Epidemiol. 1996;49:1373–1379. |
| *Minor issues:* |  |
| Page 5, line 150: Is 65 years old the age of retirement in Sweden? Maybe this could be clarified in this section. | Thank you for providing the insight. Employees in Sweden usually go for retirement after the age of 65 years, but a disability pension is an exception. Although we chose working-age women (18-65 years) for inclusion, the participants’ age at baseline range was 23-64 years (M, SD 49.63 ± 9.71). In addition, only 3 participants were included in the study aged 64 years at baseline. This has been clarified in page 5, lines 153–154, and in Table 1. |
| Page 5, line 156: In the cases where the myalgia code was used, was there any further specification to ensure that pain was located in the neck/shoulder/back? | A further explanation about why the myalgia code was used has been added (page 5, lines 158–160). |
| Page 6, line 183: The reference for the CSQ is missing. | Reference has now been added (page 6, line 188). |
| Page 6 and 7: The authors describe the CSQ and then state that they used two subscales from this questionnaire. However, when reporting the MPI the authors present its subscales separately. The same format of description should be adopted. | We agree with the Reviewer. Two subscales’ names have now been added (page 6, line 189). |
| Page 7, 228-232: Is this a validated procedure? Can a reference be provided? | A reference has been added in this regard (page 7, line 239). |
| Page 7, line 234: Can the authors be sure that the participants answered this question only considering their neck/shoulder/back pain? | This is an important question. It is hard to explain whether the participants were answering the questions only considering their neck/shoulder/back pain. However, there are mainly two reasons for which authors could make sure that the participants included in the study were experienced with neck/shoulder and/or back pain: first, the sample was chosen based on physician certificate; second, a pain figure was added in the questionnaire where the participants located their pain on the body. Despite this explanation, text about this has been added in the limitation section (page 14, lines 405–406). |
|  |  |
| RESULTS |  |
| Major issues: |  |
| Page 9, line 273: Please describe the attrition analysis in the statistical analysis section. Also, these results should be supported by an accompanying table. | We appreciate the reviewer’s suggestion, and text about attrition analysis has been added in the statistical analysis section (page 8, lines 259–260). However, we have decided to show an accompanying table under supporting information (S1 Table) instead of the manuscript. |
| Page 9, line 279: The authors state that "the group who had RTW rated less pain intensity, shorter life-long pain duration and more strongly believed in returning to work within 6 months", but this is not supported by a formal statistical test. | Thank you for the note. To see significant differences between RTW and NRTW by baseline characteristics and study variables, the *p*-value has now been added in Table 1 and text on page 9, lines 290–293. Information about analysis was added in Methods (page 8, lines 256–258). |
| Table 2. The logistic regression results should present Qui-squared statistics and R2.  Also, it would be important to have controlled for the effect of demographic variables such as age. I would expect that this variable has a strong influence on RTW. | Adjusted R^2^ and chi-squared statistics for the overall logistic regression model have been added in Table 2.  Thank you for the suggestion. Age has been controlled for in the adjusted analysis, please see in Table 2. Text about the adjusted model has been added in Methods (page 9, line 274), and in Results (page 11, lines 319–320). Adjusted analyses have been reported in the Results section. |
|  |  |
| DISCUSSION |  |
| Page 12, lines 322-324: Again, this comparisons must be based on a formal statistical test. | We agree with the reviewer’s comment. To see significant comparisons between RTW and NRTW by baseline characteristics, the *p*-value has been added in Table 1. |
