# Supplementary material for: Predictors of return to work among women with long-term neck/shoulder and/or back pain: A 1-year prospective study
Source: PLoS One. 2021 Nov 23;16(11):e0260490. doi: 10.1371/journal.pone.0260490 (PMC8610267; doi:10.1371/journal.pone.0260490)
Supplement: Attachment — Submitted filename: Response to Reviewers.docx [file pone.0260490.s006.docx]

**Authors’ response to reviews**

**Title: Predictors of return to work among women with long-term neck/shoulder and/or back pain: a 1-year prospective study**

We thank the reviewer for his/her helpful comments and feel that the changes have improved the manuscript. We have now carefully considered and revised the manuscript based on the comments that were sent to us. The following is our point-by-point reply.

| **Editor’s Comments:** |  |
| --- | --- |
| Importantly, (i) reasons to select specific subscales (CSQ and HADS) should be included in the text, (ii) as should how did the authors exclude patients with myalgia in body sites other than neck/shoulder/back; (iii) please include reference(s) that support the rationale for the cut-off set by the authors, and include this cut-off as a possible limitation in the corresponding section. (iv) the rationale for defining the clusters grouping very different variables should be clearly expressed in the text and studies validating the authors' approach should be included. | The points raised by the editor have been answered accordingly in the reviewer’s comments in the Methods section. |
| **Reviewer’s comments** |  |
| I believe all issues were satisfactorily answered, but I still have some concerns that remain, related to the methodology of the study. The reasons to select specific subscales (CSQ and HADS) were explained, but I believe these should be clearly described in the text, so that each reader can judge its adequacy. Otherwise, it may seem somewhat arbitrary. For example, the reason given in the authors’ answer to not include pain catastrophizing (non-significant results) is not acceptable. These decisions should be theory-driven.  The cut-off point for (non) return to work and the fact that participants could be performing part-time work at baseline remain an important limitation, but are mentioned in the corresponding section.  I still feel that the clusters emerging from the analysis group very different variables. I suggest that the authors cite some studies using and validating this approach, to strengthen the robustness of this approach.  Finally, I stress again that that manuscript would benefit from a thorough language revision. | Thank you for your feedback. The reasons for selecting specific subscales from CSQ and HADS have been explained on pages 6, 7 and 8, lines 198–199, 237 and 238–241, respectively.  We agree with the reviewer’s comment. Therefore, we have now mentioned this in the limitation section (page 15, lines 426–428).  To validate the approach, references have been added (page 9, line 283).  The entire manuscript has now been checked by a professional proofreader. |
| ABSTRACT |  |
| I suggest: “Cluster analyses were performed to identify one variable from each cluster to enter the regression model”. Otherwise, it is stated that cluster analyses and multiple logistic regression were performed to identify predictors. | Thank you for the suggestion. The change has been made accordingly (page 2, line 64). |
| INTRODUCTION |  |
| Page 4, line 120: “…individuals’ beliefs in their ability to work in the future and in their own influence in life as a whole are predictors of RTW”. The meaning of this sentence is not clear. | The sentence has now been clarified (page 4, lines 120–122). |
| Page 4, line 125: “Social support…was positively associated with reduced MSP and stress”. This formulation is not clear. I believe that the authors mean that social support is associated with reduced MSP and stress. A positive association would imply that more social support was associated with more stress.  Page 4, line 126: “…could increase work ability and that could in turn give an importance for RTW”. Language is still not clear in this sentence. I give the following suggestion as an example to be considered by the authors “…could increase work ability which, in turn, could contribute to RTW”. | The sentence has been corrected see on page 4, lines 124–126. |
| METHODS |  |
| Page 5, line 153: This sentence is confusing and its meaning is not clear.  Page 5, line 158: It is clear why the myalgia was code was used. But how did the authors exclude patients with myalgia in body sites other than neck/shoulder/back? In other words, how are the authors sure that only those patients complaining of myalgia in the neck/shoulder/back were included? | The sentence has been rephrased and put under exclusion criteria (page 5, lines 172–173).  All women in the study had pain in the neck, shoulder and/or back, see Table 1 (pain area). In addition, information on how the sample was selected in the study has now been moved from data collection to study sample (page 5, lines 153–156). |
| Page 6, line 188: As I understood it, all subscales were used but only these two were selected for the study. This should be clear. | This has been clarified on page 6, lines 198–199. |
| Page 7, line 228: I believe it would be of interest to clarify in the text why the anxiety subscale was not used.  Page 7, lines 230 and 239: Notwithstanding considerations made on page 8, line 246, please ponder if it would be more consistent to also present alpha values in these sections, after describing each questionnaire.  Page 7, line 237: Afterwards was correct. | Text has been added in this regard (pages 7 and 8, lines 237 and 238–241, respectively).  Considering reviewer’s comment, Cronbach’s α value of all instruments has been added after describing each questionnaire (page 6, 7 and 8, lines 202, 206, 210, 225, 233, 240–241, and 250, respectively).  Change has been done accordingly. |
| Page 8, line 251: It should be mentioned that this cut-off was set by the authors, and some studies using the same cut-off should be referenced if possible. The fact that this cut-off is a possible limitation should also be considered and mentioned in the corresponding section. | Unfortunately, we haven’t found any similar reference to support our choice of cut-off point. In the introduction, we have mentioned “The measures used vary with the population studied as well as across societies and countries, which have different healthcare systems and workers compensation regulations” (page 3, lines 106–107). However, text has been added for possible limitation on page 15, lines 426–428. |
| Page 9, line 284: I suggest adding a reference to S1 here to inform the reader about that table. | S1 Table has now been cited on page 10, line 300. |
| Page 10, line 296: To clarify this information, I suggest adding: “we chose three (plus one covariate) because…”  Page 10, line 298: Actually, the information added to is merely a repetition of what was already stated on page 9, lines 271-273. Therefore, this addition is not relevant to the content of the manuscript. | The text has been clarified, see on page 11, line 313.  Thank you for the detail. The redundant text has been deleted (page 11, lines 315–316). However, the rationale of choosing predictors has been moved to the Methods section (page 9, lines 281–285). |
| Table 2: Are the Adj R2, chi-square and p values for the adjusted or unadjusted models? Consider if both should be presented, for the reader to judge the proportion of variance explained by each model. | Thank you for the insight. We have now added *R^2^*, chi-square and *p-*values for the unadjusted model as well (see Table 2). |
| Limitations: The fact that possible litigation issues were not controlled should be mentioned as a possible limitation. | The insurance system in Sweden is centralized, meaning that if someone works in Sweden, they automatically get connected to the Swedish Social Insurance Agency (SIA), and receive sick leave benefits due to any type of diseases or disorders. The participants’ information in this study was collected from the SIA. Because all participants received sick leave benefits, we did not control for the insurance. |
